# Integrated Clinical, Biological, and Echocardiographic Factors Independently Associated With In-Hospital Mortality in Infective Endocarditis: A Single-Centre Cohort Study From Morocco

**DOI:** 10.7759/cureus.116161

**Published:** 2026-09-13

**Authors:** Tariq El Karimy, Hiba Rizkou, Joumana Elmasrioui, Mustapha Elhattaoui

**Affiliations:** 1 Department of Cardiology, Mohammed VI University Hospital, Marrakech, MAR

**Keywords:** echocardiography, infective endocarditis, in-hospital mortality, low- and middle-income countries, morocco, renal dysfunction, risk stratification, septic shock

## Abstract

Background

Infective endocarditis (IE) remains associated with substantial in-hospital mortality worldwide, with a disproportionate burden in low- and middle-income countries (LMICs). Identifying clinical, biological, microbiological, and echocardiographic factors associated with adverse outcomes may help characterize patients at increased risk of death.

Objectives

To identify clinical, biological, microbiological, and echocardiographic factors independently associated with in-hospital mortality among patients hospitalized with IE in a North African tertiary referral centre.

Methods

We conducted a retrospective observational cohort study including 260 consecutive patients admitted with definite IE according to the 2023 Duke-International Society for Cardiovascular Infectious Diseases (Duke-ISCVID) criteria, between January 2020 and April 2026. Patients were classified as survivors (n = 188) or non-survivors (n = 72). Demographic characteristics, clinical presentation, biological parameters, microbiological data, and echocardiographic findings were systematically collected. Univariable and multivariable binary logistic regression analyses were performed. Multivariable analysis used backward Wald elimination. Model discrimination was assessed using the area under the receiver operating characteristic curve, and calibration was evaluated using the Hosmer-Lemeshow test.

Results

Among 260 patients with definite IE, 188 (72.3%) survived to hospital discharge, and 72 (27.7%) died during hospitalization. The mean age was 47 ± 16 years, with a male predominance. Rheumatic heart disease was the most frequent underlying valvular condition. In multivariable analysis, septic shock was associated with increased in-hospital mortality (adjusted odds ratio (aOR), 28.45; 95% CI, 3.42-106.80; p < 0.001), as were local cardiac complications (aOR, 3.52; 95% CI, 1.42-8.72; p = 0.007), age ≥60 years (aOR, 3.84; 95% CI, 1.48-9.96; p = 0.010), vegetation diameter >15 mm (aOR, 2.50; 95% CI, 1.00-6.20; p = 0.049), male sex (aOR, 2.68; 95% CI, 1.12-6.42; p = 0.027), haemoglobin <10 g/dL (aOR, 2.80; 95% CI, 1.10-7.22; p = 0.031), and estimated glomerular filtration rate (eGFR) <30 mL/min/1.73 m² (aOR, 2.70; 95% CI, 1.03-7.01; p = 0.042).

The final model showed good discrimination, with a C-statistic of 0.84 (95% CI, 0.78-0.90), and no evidence of poor calibration according to the Hosmer-Lemeshow test (χ² = 6.42, df = 8, p = 0.60). Given the sparse distribution of septic shock among survivors (n = 2), a Firth-penalized sensitivity analysis yielded an OR of 9.80 (95% CI, 2.85-33.70), supporting the robustness of the direction of this association despite sparse-data concerns.

Conclusions

In this contemporary Moroccan cohort, septic shock, local cardiac complications, older age, vegetation diameter >15 mm, male sex, anaemia, and severe renal dysfunction were independently associated with in-hospital mortality. These findings may support early recognition of high-risk clinical features and multidisciplinary assessment, but should not be interpreted as a validated mortality prediction model. Prospective multicentre studies with internal and external validation are warranted to assess the generalizability of these findings.

## Introduction

Infective endocarditis (IE) remains a life-threatening disease associated with substantial morbidity and mortality despite advances in diagnostic imaging, antimicrobial therapy, and cardiac surgery. Its epidemiology has evolved considerably, with an increasing contribution from healthcare-associated infection, prosthetic material, and an ageing population. Recent global estimates indicate that, although age-standardized rates have declined over the past three decades, the absolute burden of endocarditis continues to increase, particularly among older adults and populations living in lower socioeconomic settings [1,2].

The burden of IE is particularly concerning in low- and middle-income countries (LMICs), where delayed diagnosis, persistent rheumatic valvular disease, differences in microbiological epidemiology, limited access to advanced imaging and cardiac surgery, and disparities in multidisciplinary care may contribute to adverse outcomes [3].

The clinical course of IE is highly heterogeneous and reflects the interaction between host characteristics, causative microorganisms, systemic complications, and the extent of cardiac involvement. Several clinical and echocardiographic features have been associated with adverse outcomes, including heart failure, renal dysfunction, neurological complications, large vegetations, and periannular extension of infection [4-7]. Echocardiography is therefore central not only to diagnosis but also to the assessment of disease severity and complications.

Current European Society of Cardiology (ESC) guidelines emphasize an integrated multidisciplinary approach to IE through dedicated Endocarditis Teams and recommend early identification of patients at increased risk of complications or death. Echocardiography provides essential information regarding valve involvement, vegetation characteristics, ventricular function, and local complications, all of which may influence prognosis and therapeutic decision-making [8].

Large international registries have contributed substantially to the understanding of contemporary IE. In particular, the ESC-EORP EURO-ENDO (European Infective Endocarditis) registry demonstrated substantial in-hospital mortality and identified several clinical, biological, and echocardiographic characteristics associated with adverse outcomes [9]. However, patients from North Africa remain relatively under-represented in international IE cohorts, and contemporary data describing prognostic factors among Moroccan patients are limited.

Identifying factors associated with mortality in this setting may provide complementary information to international cohorts and help characterize patients at increased risk of adverse outcomes. This is particularly relevant in tertiary referral centres, where patients may present with advanced infection and complex cardiac complications.

Accordingly, this study aimed to identify independent clinical, biological, microbiological, and echocardiographic factors associated with in-hospital mortality among patients with definite IE admitted to a tertiary cardiology centre in Morocco.

## Materials and methods

Study design and setting

This retrospective observational cohort study was conducted in the Department of Cardiology at Mohammed VI University Hospital, a tertiary referral centre in Marrakech, Morocco. The study included consecutive patients admitted between January 2020 and April 2026 with definite IE.

Study population

All consecutive patients admitted between January 2020 and April 2026 with a diagnosis of definite IE according to the 2023 Duke-International Society for Cardiovascular Infectious Diseases (Duke-ISCVID) criteria were screened [10]. Diagnoses were adjudicated retrospectively by a panel of three cardiologists, who independently reviewed each patient's clinical, microbiological, and echocardiographic data. Cases of diagnostic uncertainty or disagreement were resolved by consensus discussion among the three reviewers.

Of 283 patients screened, 23 were excluded: 14 because of incomplete medical records precluding assessment of the primary outcome and nine because the diagnosis of IE was not retained. The final study population comprised 260 patients (Figure 1).

**Figure 1:**
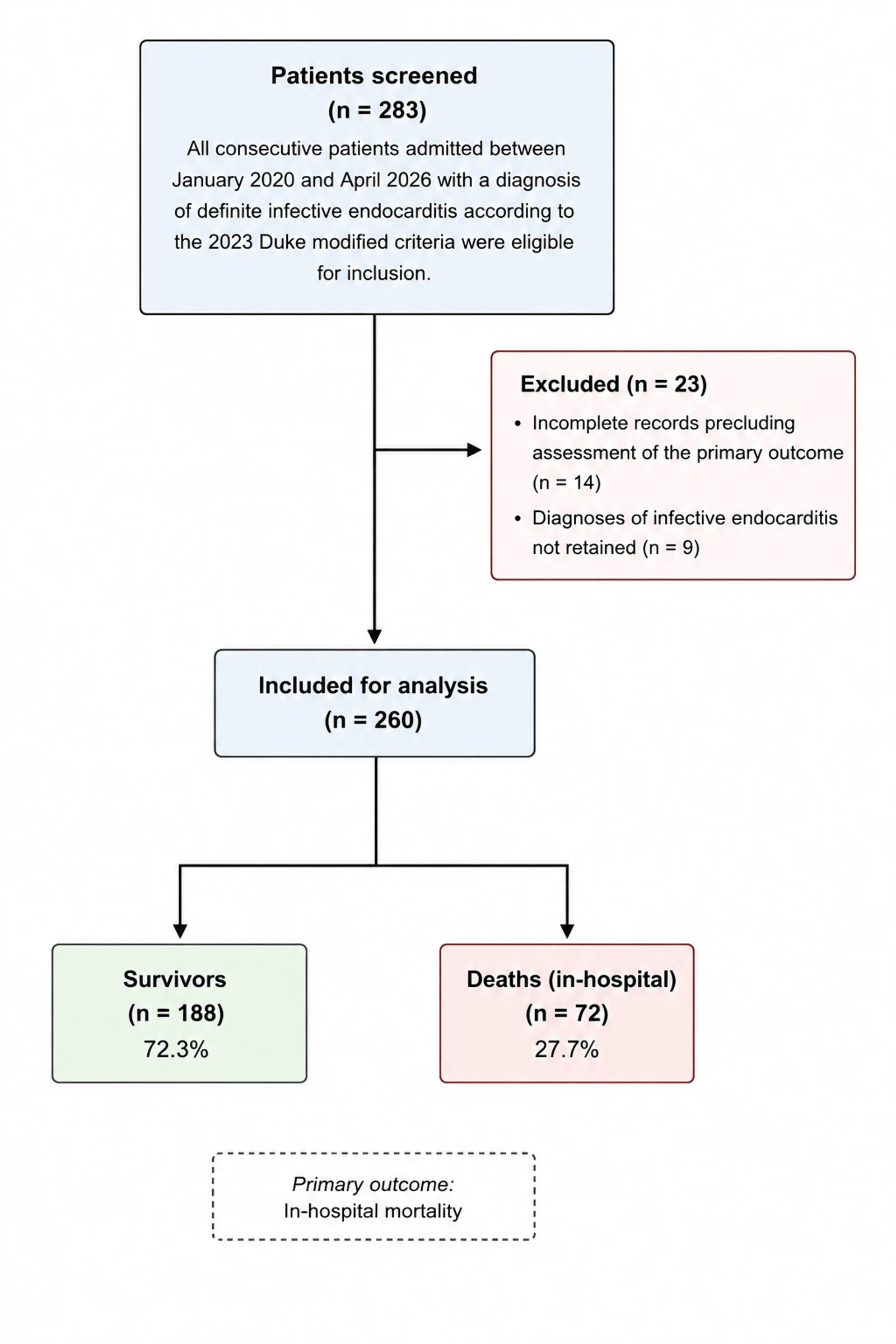
Flowchart of patient selection and study cohort. Flowchart depicting the selection of patients included in the study. Among 283 consecutive patients screened between January 2020 and April 2026 for definite infective endocarditis according to the 2023 Duke-International Society for Cardiovascular Infectious Diseases (Duke-ISCVID) criteria [10], 23 patients were excluded because of incomplete medical records precluding assessment of the primary outcome (n = 14) or because the diagnosis of infective endocarditis was not retained (n = 9). A total of 260 patients were included in the final analysis, comprising 188 survivors (72.3%) and 72 patients who died during hospitalization (27.7%). The primary outcome was all-cause in-hospital mortality.

Paediatric patients (<16 years) were not included in this cohort, which was drawn exclusively from an adult cardiology department. Patients transferred to another institution after diagnosis but before hospital discharge were excluded from the assessment of the primary outcome when in-hospital mortality could not be ascertained beyond the index admission. Readmissions for IE during the study period were counted as a single episode indexed to the first qualifying admission to avoid duplicate inclusion of the same clinical episode.

Data collection and definitions

Clinical, biological, microbiological, and echocardiographic data were retrospectively extracted from medical records using a standardized data collection form. Three physicians reviewed the medical records. Baseline clinical, biological, and echocardiographic data were collected at the time of admission, prior to the initiation or completion of antimicrobial and surgical treatment, to characterize disease severity at presentation.

Clinical complications occurring at any point during hospitalization, including septic shock, local cardiac complications, and neurological events, were recorded regardless of their timing relative to admission and were therefore considered complications observed during the index hospitalization rather than strictly baseline characteristics.

Anaemia was defined as a haemoglobin concentration <10 g/dL. Severe renal dysfunction was defined as an estimated glomerular filtration rate (eGFR) <30 mL/min/1.73 m², calculated using the Chronic Kidney Disease Epidemiology Collaboration (CKD-EPI) equation. These thresholds were used as predefined analytical categories and were not intended to represent universal diagnostic thresholds.

Demographic characteristics, cardiovascular comorbidities, clinical presentation, microbiological findings, echocardiographic characteristics, laboratory parameters, and treatment-related variables were collected.

Data were collected from the available medical records. Patients with missing data for the primary outcome were excluded a priori. No imputation was performed for missing covariate data. Complete data for all candidate variables were available in all 260 included patients; therefore, the multivariable analysis was performed on the full study cohort.

Echocardiographic assessment

All patients underwent transthoracic echocardiography (TTE) at admission, performed and interpreted by attending cardiologists with expertise in echocardiography. Transesophageal echocardiography (TEE) was additionally performed in 248 of 260 patients (95.4%) when clinically indicated, notably in cases of inconclusive TTE findings, suspected prosthetic valve involvement, or clinical suspicion of local cardiac complications.

When both TTE and TEE were available, vegetation size and local cardiac complications were determined using TEE findings, given its superior sensitivity for these features. All echocardiographic studies were independently reviewed by a second cardiologist blinded to clinical outcome. Discordant measurements or interpretations were resolved by joint re-review with a third senior cardiologist.

Technically limited or non-diagnostic examinations were repeated when clinically feasible. If repeat imaging was not possible, the best available assessment was retained, which may have introduced some measurement imprecision. Echocardiographic assessment included valve type (native or prosthetic), valve(s) involved, vegetation presence, maximal vegetation diameter, number of vegetations, left ventricular ejection fraction (LVEF), and local cardiac complications. Local cardiac complications were defined as periannular abscess, pseudoaneurysm, fistula, leaflet perforation, or prosthetic valve dehiscence.

The maximal vegetation diameter was recorded from the echocardiographic examination. A large vegetation was defined as a maximal diameter >15 mm, consistent with thresholds used in prior echocardiographic studies of embolic risk in IE [5,6]. This threshold was used as a predefined analytical category and should not be interpreted as a universal surgical threshold.

Representative echocardiographic findings from the study cohort are presented in Figure 2.

**Figure 2:**
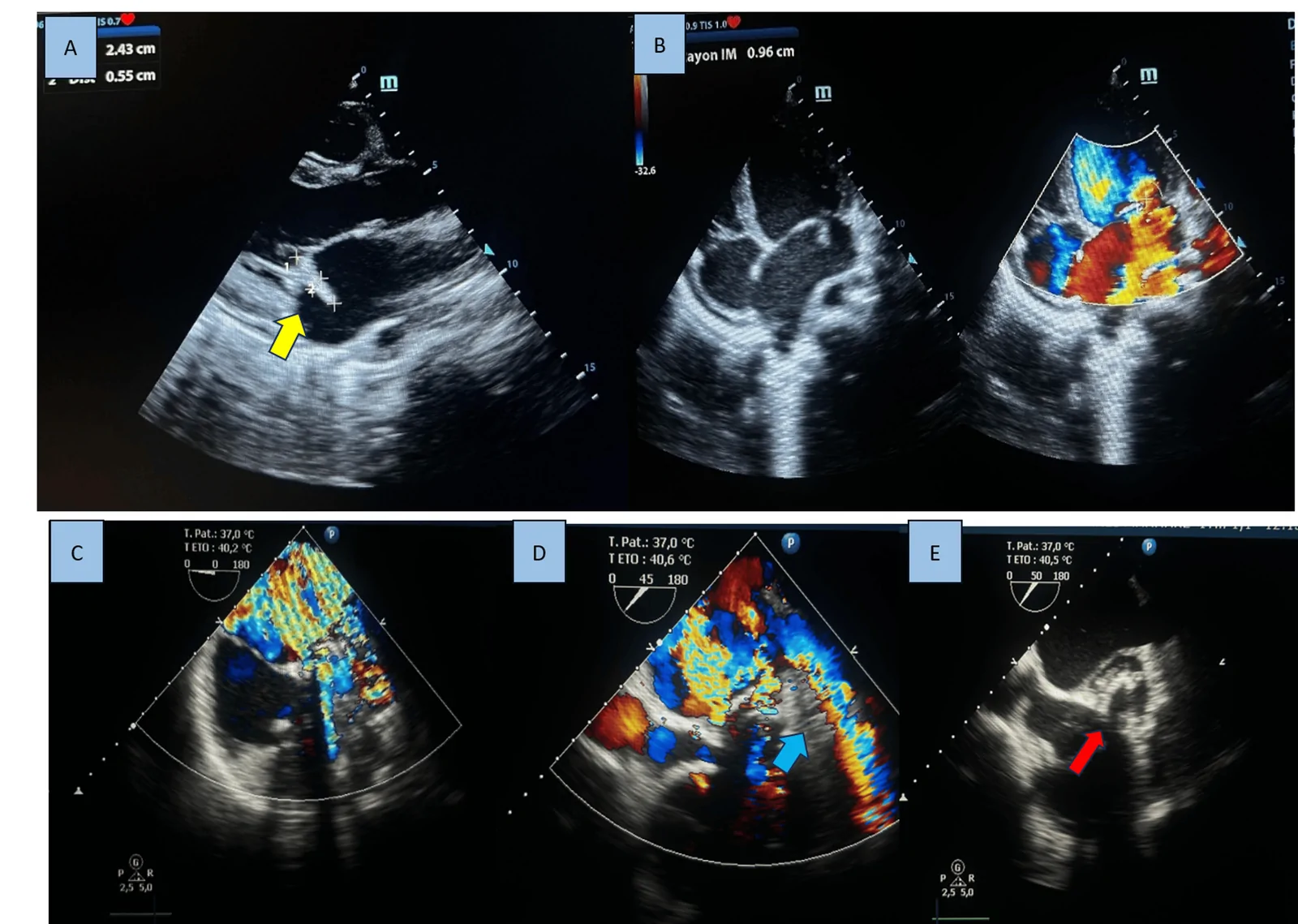
Representative echocardiographic findings in infective endocarditis. (A) TTE showing a large mitral valve vegetation, measuring 2.43 cm in maximal length (yellow arrow). (B) TEE with colour Doppler demonstrating severe mitral regurgitation. (C-D) TEE demonstrating dehiscence of a prosthetic mitral valve (blue arrow). (E) TEE demonstrating an aortic perivalvular abscess (red arrow). Abbreviations: TTE, transthoracic echocardiography; TEE, transesophageal echocardiography.

Management and outcome

Antimicrobial therapy was administered according to contemporary clinical recommendations and adapted to the microbiological findings and clinical status of each patient. Surgical indications were assessed by the treating multidisciplinary team according to clinical, microbiological, and echocardiographic characteristics.

Three sets of blood cultures were obtained before initiation of antibiotic therapy, in accordance with contemporary guideline recommendations. Empirical intravenous antibiotic therapy was initiated immediately after blood cultures were collected. Most patients received an amoxicillin- or ceftriaxone-based regimen combined with gentamicin for community-acquired native-valve or late prosthetic-valve IE, whereas vancomycin-based therapy combined with gentamicin and rifampicin was used for early prosthetic-valve or healthcare-associated IE. Antibiotic therapy was subsequently adapted according to the identified microorganism and antimicrobial susceptibility testing. In patients undergoing cardiac surgery, antimicrobial therapy was continued postoperatively and subsequently adjusted according to intraoperative microbiological findings and susceptibility results when available. The mean duration of antibiotic therapy was 6 ± 2 weeks.

The primary outcome was all-cause in-hospital mortality, defined as death occurring during the index hospitalization.

Among patients who underwent surgery, the time from admission to surgery was recorded. Deaths occurring before planned or considered surgical intervention were identified separately to account for the potential influence of survival time on the observed association between surgery and mortality.

Statistical analysis

Continuous variables were expressed as mean ± standard deviation or median with interquartile range, as appropriate. Categorical variables were presented as frequencies and percentages.

Comparisons between survivors and non-survivors were performed using Student's t-test or Mann-Whitney U test for continuous variables, as appropriate, and the chi-square test or Fisher's exact test for categorical variables, as appropriate.

Univariable binary logistic regression analyses were performed to estimate crude odds ratios (ORs) and 95% confidence intervals (CIs) for the association between candidate variables and in-hospital mortality.

For multivariable analysis, 20 candidate variables were initially considered: septic shock, local cardiac complications, age ≥60 years, vegetation diameter >15 mm, male sex, haemoglobin <10 g/dL, eGFR <30 mL/min/1.73 m², hypertension, right heart failure, left heart failure, neurological complications, fever >39°C, New York Heart Association (NYHA) class III-IV dyspnoea, multiple vegetations, staphylococcal infection, white blood cell count >12,000/mm³, platelet count <150,000/mm³, C-reactive protein >100 mg/L, and procalcitonin >1 ng/mL, together with prosthetic valve IE.

LVEF <40% was not included in the multivariable analysis because of the very small number of patients meeting this criterion (n = 6). Urea >1 g/L and creatinine >15 mg/L were also excluded a priori because of their collinearity with eGFR; eGFR <30 mL/min/1.73 m² was retained as the summary measure of renal dysfunction.

The multivariable model was constructed using binary logistic regression with backward Wald elimination. Variables with Wald p > 0.05 were successively removed until the final model was obtained. The final model retained seven variables: septic shock, local cardiac complications, age ≥60 years, vegetation diameter >15 mm, male sex, haemoglobin <10 g/dL, and eGFR <30 mL/min/1.73 m². Adjusted odds ratios (aORs) with 95% CIs were reported, with a two-sided p < 0.05 considered statistically significant.

As septic shock was present in only two survivors, a Firth-penalized logistic regression sensitivity analysis was additionally performed for this sparse exposure. The resulting estimate was compared with the conventional logistic regression estimate.

Model discrimination was assessed using the area under the receiver operating characteristic curve (AUC), with its 95% CI. Model calibration was assessed using the Hosmer-Lemeshow goodness-of-fit test.

All analyses were performed using Microsoft Excel (Microsoft Corporation, Redmond, WA) for data management and IBM SPSS Statistics version 29 (IBM Corp., Armonk, NY) for statistical analysis.

Ethical considerations

The study was conducted in accordance with the Declaration of Helsinki. Given the retrospective nature of the study and the use of anonymized data, informed consent was waived by the Biomedical Research Ethics Committee of the Faculty of Medicine and Pharmacy of Marrakech, Cadi Ayyad University (approval reference: CE-FMPM/2020 - 17/2020).

## Results

Study population and baseline characteristics

Among 260 patients with definite infective endocarditis, 188 (72.3%) survived to hospital discharge and 72 (27.7%) died during hospitalization. The mean age was 47 ± 16 years, with a male predominance. Rheumatic heart disease was the most frequent underlying valvular condition.

Regarding valve type, native-valve endocarditis was present in 232 patients (89.23%), whereas prosthetic-valve endocarditis accounted for 28 cases (10.77%). Mitral valve involvement was observed in 87 patients (33.46%), aortic valve involvement in 80 (30.77%), tricuspid involvement in six (2.31%), combined mitro-aortic involvement in 55 (21.15%), and combined mitro-aortic-tricuspid involvement in four (1.54%).

Compared with survivors, patients who died during hospitalization were older and more frequently male. Several clinical characteristics, including hypertension, right-sided heart failure, local cardiac complications, septic shock, fever >39°C, and NYHA functional class III-IV dyspnoea, were more frequent among non-survivors. The detailed comparison between survivors and non-survivors is presented in Table 1.

**Table 1:** Univariable analysis of clinical, microbiological, echocardiographic, and biological factors associated with in-hospital mortality. Abbreviations: CI, confidence interval; CRP, C-reactive protein; eGFR, estimated glomerular filtration rate; Hb, haemoglobin; IE, infective endocarditis; LVEF, left ventricular ejection fraction; NYHA, New York Heart Association; OR, odds ratio; WBC, white blood cell count. * Local cardiac complications were defined as periannular abscess, pseudoaneurysm, fistula, leaflet perforation, or prosthetic valve dehiscence. Statistical note: ORs and corresponding p-values were obtained from univariable binary logistic regression analyses. ORs represent the odds of in-hospital mortality associated with each variable.

Variable	Survivors (n = 188)	Deceased (n = 72)	OR (95% CI)	p-value
Demographic and comorbidity variables				
Age ≥60 years	24 (12.8%)	24 (33.3%)	3.42 (1.78–6.55)	<0.001
Male sex	76 (40.4%)	48 (66.7%)	2.95 (1.67–5.21)	<0.001
Hypertension	21 (11.2%)	20 (27.8%)	3.06 (1.54–6.08)	0.001
Diabetes	23 (12.2%)	10 (13.9%)	1.16 (0.52–2.57)	0.720
Prosthetic valve IE	16 (8.5%)	12 (16.7%)	2.15 (0.96–4.80)	0.058
Clinical presentation and complications				
Right heart failure	38 (20.2%)	30 (41.7%)	2.82 (1.57–5.08)	<0.001
Left heart failure	22 (11.7%)	16 (22.2%)	2.16 (1.06–4.39)	0.032
Local cardiac complications*	36 (19.1%)	36 (50.0%)	4.22 (2.35–7.60)	<0.001
Septic shock	2 (1.1%)	20 (27.8%)	35.77 (8.10–158.04)	<0.001
Neurological complication	22 (11.7%)	19 (26.4%)	2.70 (1.36–5.38)	0.004
Fever >39°C	106 (56.4%)	60 (83.3%)	3.87 (1.95–7.66)	<0.001
Dyspnoea (NYHA III–IV)	36 (19.1%)	28 (38.9%)	2.69 (1.48–4.88)	<0.001
Echocardiographic variables				
LVEF <40%	2 (1.1%)	4 (5.6%)	5.47 (0.98–30.55)	0.052
Vegetation >15 mm	20 (10.6%)	20 (27.8%)	3.23 (1.61–6.46)	<0.001
Multiple vegetations	84 (44.7%)	48 (66.7%)	2.48 (1.40–4.37)	0.002
Mitral valve	63 (33.5%)	24 (33.3%)	0.99 (0.56–1.76)	0.978
Aortic valve	60 (31.9%)	20 (27.8%)	0.82 (0.45–1.50)	0.52
Mitro-aortic	38 (20.2%)	17 (23.6%)	1.22 (0.64–2.34)	0.55
Microbiological variables				
Staphylococcal infection	14 (7.4%)	12 (16.7%)	2.49 (1.09–5.67)	0.027
Streptococcal infection	32 (17.0%)	10 (13.9%)	0.79 (0.36–1.70)	0.539
Biological variables				
WBC count >12,000/mm³	53 (28.2%)	54 (75.0%)	7.64 (4.11–14.22)	<0.001
Hb <10 g/dL	56 (29.8%)	54 (75.0%)	7.07 (3.81–13.12)	<0.001
Platelets <150,000/mm³	14 (7.4%)	24 (33.3%)	6.21 (2.99–12.93)	<0.001
CRP >100 mg/L	55 (29.3%)	40 (55.6%)	3.02 (1.72–5.30)	<0.001
Procalcitonin >1 ng/mL	11 (5.9%)	19 (26.4%)	5.40 (2.43–12.47)	<0.001
Urea >1 g/L	8 (4.3%)	16 (22.2%)	6.43 (2.61–15.81)	<0.001
Creatinine >15 mg/L	9 (4.8%)	20 (27.8%)	7.65 (3.29–17.81)	<0.001
eGFR <30 mL/min/1.73 m²	11 (5.9%)	18 (25.0%)	5.36 (2.39–12.05)	<0.001
Treatment				
Surgery performed	71 (37.8%)	22 (30.6%)	0.73 (0.41–1.30)	0.278

Clinical and echocardiographic factors associated with mortality

In univariable analysis, several clinical and echocardiographic characteristics were significantly associated with in-hospital mortality (Table 1). Patients aged ≥60 years had higher odds of death than younger patients (OR, 3.42; 95% CI, 1.78-6.55; p < 0.001). Male sex was also associated with increased mortality (OR, 2.95; 95% CI, 1.67-5.21; p < 0.001).

Among clinical complications, local cardiac complications were significantly more frequent among non-survivors and were associated with more than a four-fold increase in the odds of death (OR, 4.22; 95% CI, 2.35-7.60; p < 0.001). Right heart failure was also associated with mortality (OR, 2.82; 95% CI, 1.57-5.08; p < 0.001). Septic shock was strongly associated with in-hospital mortality (OR, 35.77; 95% CI, 8.10-158.04; p < 0.001). Neurological complications, fever >39°C, and NYHA class III-IV dyspnoea were also significantly associated with mortality.

Regarding echocardiographic characteristics, vegetation diameter >15 mm was associated with increased mortality (OR, 3.23; 95% CI, 1.61-6.46; p < 0.001). Multiple vegetations were similarly associated with mortality (OR, 2.48; 95% CI, 1.40-4.37; p = 0.002). LVEF <40% was not statistically significant (p = 0.052).

Valve involvement was not significantly associated with in-hospital mortality: mitral valve involvement (OR, 0.99; 95% CI, 0.56-1.76; p = 0.978), aortic valve involvement (OR, 0.82; 95% CI, 0.45-1.50; p = 0.52), and combined mitro-aortic involvement (OR, 1.22; 95% CI, 0.64-2.34; p = 0.55) showed no significant relationship with outcome. Tricuspid valve involvement (n = 6, 2.3%) and combined mitro-aortic-tricuspid involvement (n = 4, 1.5%) were too infrequent for meaningful statistical comparison.

Biological and microbiological factors associated with mortality

Several biological markers of systemic inflammation and organ dysfunction were strongly associated with in-hospital mortality (Table 1). Leukocytosis, defined as a white blood cell count >12,000/mm³, was strongly associated with mortality (OR, 7.64; 95% CI, 4.11-14.22; p < 0.001). Haemoglobin <10 g/dL was similarly associated with markedly increased odds of death (OR, 7.07; 95% CI, 3.81-13.12; p < 0.001), as was thrombocytopenia (OR, 6.21; 95% CI, 2.99-12.93; p < 0.001).

CRP >100 mg/L was associated with mortality (OR, 3.02; 95% CI, 1.72-5.30; p < 0.001), while procalcitonin >1 ng/mL was also significantly associated with death (OR, 5.40; 95% CI, 2.43-12.47; p < 0.001).

Markers of renal dysfunction were consistently associated with mortality. Urea >1 g/L, creatinine >15 mg/L, and eGFR <30 mL/min/1.73 m² were all significantly more frequent among non-survivors.

Staphylococcal infection was also associated with mortality in univariable analysis (OR, 2.49; 95% CI, 1.09-5.67; p = 0.027), whereas streptococcal infection was not significantly associated with mortality.

Independent factors associated with in-hospital mortality

Twenty prespecified candidate variables were initially entered into the multivariable model. After backward Wald elimination, seven variables remained independently associated with in-hospital mortality (Table 2).

**Table 2:** Multivariable logistic regression analysis of independent factors associated with in-hospital mortality. Abbreviations: CI, confidence interval; eGFR, estimated glomerular filtration rate; Hb, haemoglobin; OR, odds ratio. Note: Twenty prespecified candidate variables were considered for multivariable analysis. The final model was obtained using backward Wald elimination. Only variables retained in the final model are shown. Adjusted ORs and 95% CIs represent the association with in-hospital mortality after multivariable adjustment.

Variable	Adjusted OR	95% CI	p-value
Septic shock	28.45	3.42–106.8	<0.001
Local cardiac complications	3.52	1.42–8.72	0.007
Age ≥60 years	3.84	1.48–9.96	0.010
Vegetation >15 mm	2.50	1.00–6.20	0.049
Male sex	2.68	1.12–6.42	0.027
Hb <10 g/dL	2.80	1.10–7.22	0.031
eGFR <30 mL/min/1.73 m²	2.70	1.03–7.01	0.042

Septic shock was independently associated with in-hospital mortality (aOR, 28.45; 95% CI, 3.42-106.80; p < 0.001). Local cardiac complications were also independently associated with mortality (aOR, 3.52; 95% CI, 1.42-8.72; p = 0.007).

Age ≥60 years remained independently associated with mortality (aOR, 3.84; 95% CI, 1.48-9.96; p = 0.010). A vegetation diameter >15 mm was also independently associated with mortality (aOR, 2.50; 95% CI, 1.00-6.20; p = 0.049).

Male sex was independently associated with mortality (aOR, 2.68; 95% CI, 1.12-6.42; p = 0.027). Anaemia, defined as haemoglobin <10 g/dL, remained independently associated with mortality (aOR, 2.80; 95% CI, 1.10-7.22; p = 0.031), as did severe renal dysfunction defined by eGFR <30 mL/min/1.73 m² (aOR, 2.70; 95% CI, 1.03-7.01; p = 0.042).

Given the sparse distribution of septic shock among survivors (n = 2), a Firth-penalized logistic regression was performed as a sensitivity analysis. The resulting odds ratio was 9.80 (95% CI, 2.85-33.70), and the association remained statistically significant, consistent with the direction of the conventional logistic regression estimate.

The final model showed good discrimination, with a C-statistic (area under the receiver operating characteristic curve) of 0.84 (95% CI, 0.78-0.90). The Hosmer-Lemeshow test did not indicate evidence of poor model calibration (χ² = 6.42, df = 8, p = 0.60).

Overall, the multivariable analysis identified systemic severity, local cardiac complications, echocardiographic vegetation burden, and markers of host vulnerability as independent factors associated with in-hospital mortality.

## Discussion

In this contemporary single-centre cohort of 260 patients with definite IE, in-hospital mortality was 27.7%. Independent associations with mortality involved systemic severity, local cardiac extension, echocardiographic vegetation burden, and host vulnerability. These findings support an integrated approach to risk assessment in IE, particularly in a tertiary-care setting.

Systemic severity and local cardiac complications

Septic shock showed the strongest association with mortality in our cohort. This finding is consistent with the established prognostic importance of haemodynamic instability and uncontrolled infection in IE [8,9]. As only two survivors had septic shock, the conventional estimate was imprecise. The Firth-penalized sensitivity analysis produced a lower but still statistically significant estimate, supporting the direction of the association while emphasizing that its magnitude should be interpreted cautiously. Clinically, septic shock should prompt immediate reassessment of haemodynamic status, organ dysfunction, infection control, and potential indications for urgent intervention.

Local cardiac complications, including abscess, pseudoaneurysm, fistula, leaflet perforation, and prosthetic valve dehiscence, were also independently associated with mortality. These lesions reflect extension of infection beyond the valve and are recognized as important manifestations of locally uncontrolled infection in current guidelines [8]. Their association with mortality is also consistent with international IE cohorts [9]. Importantly, this observational association should not be interpreted as evidence that surgery itself necessarily reduces mortality in every patient; surgical decisions remain individualized according to the overall clinical context.

Echocardiographic disease burden

A vegetation diameter >15 mm remained independently associated with mortality. Larger vegetations may reflect a greater burden of infected tissue and are associated with an increased risk of embolic complications [5,6,8]. The finding is also broadly consistent with EURO-ENDO, in which vegetation length >10 mm was associated with mortality [9]. However, the >15-mm threshold in the present study was a predefined analytical category rather than a universal surgical threshold, and the borderline statistical significance and confidence interval close to unity warrant cautious interpretation. Measurement variability between TTE and TEE may also have contributed to imprecision.

Multiple vegetations were associated with mortality in univariable analysis but did not remain independently associated after adjustment. This may reflect overlap with vegetation size and systemic severity rather than an absence of clinical relevance. Similarly, the absence of a significant association between individual valve location and mortality should be interpreted in the context of the distribution of valve involvement and the relatively small numbers of some valve categories.

Age, sex, anaemia, and renal dysfunction

Age ≥60 years was independently associated with mortality, consistent with previous studies identifying older age as an adverse prognostic factor in IE [11,12]. Although the overall cohort was relatively young, likely reflecting the continued burden of rheumatic valvular disease in this setting, older patients may have lower physiological reserve and a greater burden of comorbidity.

Male sex was also independently associated with mortality. Possible explanations include differences in comorbidity, disease characteristics, healthcare-seeking behaviour, or biological factors, but these hypotheses were not assessed in our dataset. Accordingly, this finding should be considered an epidemiological association requiring confirmation in other cohorts.

Anaemia and severe renal dysfunction remained independently associated with mortality. Anaemia may reflect inflammation, chronic disease, renal dysfunction, nutritional factors, haemodilution, or the acute infectious process; previous studies have similarly associated anaemia with adverse outcomes in IE [13]. Renal dysfunction is a well-established adverse prognostic feature in IE and is consistent with findings from EURO-ENDO and previous studies [7,9]. As these variables were assessed retrospectively, their temporal relationship with the infectious episode cannot be established.

Other biological and microbiological factors

Leukocytosis, thrombocytopenia, elevated CRP, and procalcitonin were associated with mortality in univariable analyses but did not remain independently associated after adjustment. Their attenuation may reflect overlap with systemic severity and other stronger predictors rather than a lack of clinical relevance. They nevertheless remain useful components of the initial assessment of disease severity.

Staphylococcal infection was also associated with mortality in univariable analysis but not after adjustment. This may reflect its relationship with systemic severity and cardiac complications. The relatively limited number of microbiologically characterized infections also restricts the precision of pathogen-specific comparisons.

Antibiotic therapy and surgery

In our cohort, empirical intravenous antibiotic therapy was initiated immediately after collection of three sets of blood cultures and subsequently adapted according to microbiological identification and susceptibility testing. Treatment was continued postoperatively in patients undergoing surgery and adjusted when additional microbiological information became available. The mean duration of treatment was 6 ± 2 weeks. These features are relevant when interpreting surgical outcomes because antimicrobial treatment and surgery are complementary components of IE management rather than independent therapeutic strategies. Current guidelines emphasize timely surgery for selected patients with heart failure, uncontrolled infection, or prevention of embolic events [8].

The absence of a significant unadjusted association between surgery and mortality should therefore not be interpreted as evidence against a surgical benefit. Surgical treatment is strongly influenced by disease severity and indication, creating confounding by indication. In addition, the median interval from admission to surgery was seven days, and eight patients died before planned or considered surgery, illustrating the potential contribution of survivor and immortal-time bias. Causal conclusions regarding the effect of surgery cannot therefore be drawn from this analysis.

Comparison with international data

The 27.7% in-hospital mortality observed in this cohort was numerically higher than the 17.1% reported in EURO-ENDO [9]. Direct comparison should nevertheless be cautious because of differences in referral patterns, case mix, access to cardiac surgery, healthcare organization, and study populations. As a tertiary referral-centre cohort, our population may have included a disproportionate number of patients with advanced or complicated disease. Despite these differences, the overall pattern of prognostic factors was broadly concordant with international cohorts and provides contemporary data from a North African population that remains under-represented in large IE studies.

Clinical implications

The findings support multidimensional assessment of patients with IE, incorporating haemodynamic status, local cardiac complications, echocardiographic vegetation burden, renal function, haemoglobin, and demographic characteristics. These variables are readily available through routine clinical, laboratory, and echocardiographic assessment and may help identify patients requiring particularly close monitoring and early multidisciplinary discussion. However, they should be regarded as associated risk factors rather than as a validated mortality score.

Limitations

This study has several limitations. First, its retrospective, single-centre design limits generalizability and introduces potential selection and information bias, including referral bias inherent to a tertiary centre. Second, the 72 deaths among 260 patients limited statistical precision for some estimates, particularly septic shock, for which only two survivors were exposed. The lower Firth-penalized estimate further indicates that the conventional effect size should be interpreted cautiously.

Third, 20 candidate variables were initially considered for multivariable modelling, corresponding to an events-per-variable ratio of approximately 3.6 for the initial model. The use of backward Wald elimination may therefore have introduced variable-selection instability and optimistic effect estimates. No internal validation, such as bootstrap resampling, was performed, and external validation in independent cohorts is required before these findings can support a formal prognostic score.

Fourth, treatment decisions, particularly surgery, were not randomized and were subject to confounding by indication. The timing of surgery relative to death further limits causal interpretation of the surgery-mortality association. Fifth, microbiological findings may have been influenced by previous antibiotic exposure, and the number of culture-positive infections limited pathogen-specific analyses. Finally, only in-hospital mortality was assessed; the identified associations cannot necessarily be extrapolated to long-term mortality or late complications.

Overall interpretation

In this contemporary Moroccan tertiary-centre cohort, in-hospital mortality from IE was substantial and was independently associated with systemic severity, local cardiac extension, vegetation burden, and markers of host vulnerability. These findings support multidimensional clinical and echocardiographic risk assessment while highlighting the need for prospective multicentre validation before these associations can be incorporated into a formal prognostic tool.

## Conclusions

IE remains associated with substantial in-hospital mortality in Morocco. In this single-centre cohort, septic shock, local cardiac complications, vegetation diameter >15 mm, older age, male sex, anaemia, and severe renal dysfunction were independently associated with in-hospital mortality. These findings may support early recognition of high-risk features and prompt multidisciplinary assessment. However, prospective multicentre studies are required to validate these associations before they can be incorporated into formal risk-stratification tools.
